# Comment on Yoo et al. Amylin Protein Expression in the Rat Brain and Neuro-2a Cells. *Int. J. Mol. Sci.* 2022, *23*, 4348

**DOI:** 10.3390/ijms24021058

**Published:** 2023-01-05

**Authors:** Tayla A. Rees, Debbie L. Hay, Christopher S. Walker

**Affiliations:** 1 School of Biological Sciences, University of Auckland, Auckland 1010, New Zealand; 2 Maurice Wilkins Centre for Molecular Biodiscovery, University of Auckland, Auckland 1010, New Zealand; 3 Department of Pharmacology and Toxicology, University of Otago, Dunedin 9016, New Zealand

We read with great interest the recent article by Yoo and colleagues [1] titled “Amylin Protein Expression in the Rat Brain and Neuro-2a Cells”. This study investigated the distribution of amylin-like immunoreactivity throughout the rat brain, a subject of growing clinical interest. One of the major findings was the widespread expression of amylin in regions of the rat brain linked to both metabolism and pain. However, caution needs to be taken when interpreting the clinical translatability and impact of these findings due to limitations in the methodology.

The specific detection of amylin expression in nervous tissue is challenging due to the abundance of calcitonin gene-related peptides (CGRP). Some mRNA and antibody-based tools are unable to sufficiently distinguish between these two related peptides [2,3,4,5]. We recently highlighted this issue by investigating the cross-reactivity between amylin and CGRP for several anti-amylin antibodies which had previously been used in human and rodent nervous tissues. We observed that all of the anti-amylin antibodies that could effectively detect amylin also displayed variable cross-reactivity with physiologically relevant concentrations of rodent αCGRP [6]. We then used an amylin-specific antibody to show that any amylin-like staining in human trigeminal ganglia neurons was likely due to cross-reactivity with CGRP [7]. These data, together with historical reports, build a compelling argument that researchers must consider the potential contribution of CGRP to immunoreactivity detected using anti-amylin antibodies in the nervous system.

Yoo and colleagues acknowledge the significant complication CGRP cross-reactivity has caused for the identification of amylin in the nervous system. However, it is unclear how this issue has been addressed in this study. The potential contribution of CGRP to their observed amylin-like immunoreactivity must be considered because CGRP mRNA and/or protein are widespread in the brain and have been described in many of the regions investigated [8]. For example, the cerebellar and brainstem amylin immunoreactivity patterns presented by Yoo and colleagues in figure 1 strongly resemble previous reports of CGRP in these regions [9,10]. In contrast, amylin mRNA and protein have much more restricted expression, such as in the preoptic area of the hypothalamus [11,12,13,14]. Although Yoo and colleagues do not state the exact region of the hypothalamus investigated, genuine amylin staining could be present in this location. Interestingly, comparatively few anti-CGRP antibodies appear to exhibit cross-reactivity with amylin [15]. This suggests that the cross-reactivity of anti-CGRP antibodies with amylin is less likely to be a confounding consideration in interpreting CGRP expression in the nervous system. 

An alternative conclusion to the immunoreactivity observed by Yoo and colleagues being the detection of amylin is that some or all of the immunoreactivity is instead cross-reactive detection of CGRP. This is supported by the author’s discussion, which notes the similarity of their anti-amylin antibody to T4157, which is known to be highly cross-reactive with CGRP [6,16]. Given this, the findings of this study should be interpreted cautiously with CGRP in mind.
